# High frequency electrocoagulation resection effect analysis and prognosis observation in the treatment of patients with gastric polyps under painless gastroscopy

**DOI:** 10.1097/MD.0000000000037027

**Published:** 2024-02-09

**Authors:** Xiaomei Chen, Dandan Zhang, Mei Chen

**Affiliations:** a Department of Nursing, The Fifth People’s Hospital of Wuhu City, Wuhu, Anhui, People’s Republic of China.

**Keywords:** aortic regurgitation, arterial switch, congenital heart disease, transposition of the great arteries

## Abstract

To explore high frequency electrocoagulation resection effect in treatment of patients with gastric polyps under painless gastroscopy. Sixty-four patients with gastric polyps were randomly divided into experimental group (32 cases) and control group (32 cases). Experimental group received basic treatment drugs for 8 weeks, and then treated with painless gastroscope high-frequency electrocoagulation resection. Control group was also given basic treatment drugs for 8 weeks, and then received high-frequency electrocoagulation resection under ordinary gastroscope. The patients in both groups were given rabeprazole sodium enteric coated capsules for 4 weeks. The improvement of symptom score, postoperative gastric mucosal healing and comprehensive curative effect of the 2 groups were observed after treatment. The patients with polyps cured under gastroscopy were subjected to a 6-month follow-up period during which gastroscopy was performed to assess the recurrence of polyps. Symptom scores comparison after treatment showed that experimental group had obvious advantages in improving epigastric fullness, fatigue and loose stool in patients with gastric polyps (*P* < .01 or *P* < .05). Gastric mucosa healing in experimental group was better at 2 weeks after operation (*P* < .05), showing no difference 4 weeks after operation (*P* > .05). Comprehensive curative effect comparison showed that the experimental group was better (*P* < .01), showing no difference in long-term efficacy (*P* > .05). In treating patients with gastric polyps, painless endoscopic high-frequency electrocoagulation resection effect is better, which not only promotes postoperative rehabilitation in patients but also reduces complications incidence, demonstrating a high level of safety. Therefore, it is highly recommended for widespread adoption and application.

## 1. Introduction

Gastric polyps are benign growths that protrude into the stomach and originate from the gastric mucosa or submucosa. The precise causes and mechanisms responsible for gastric polyps remain partially unknown. Notably, some types of gastric polyps, particularly adenomatous and proliferative polyps, are considered precancerous and pose a risk of developing into gastric cancer.^[1,2]^ Patients with gastric polyps typically do not exhibit specific clinical symptoms. The gold standard for diagnosis involves gastroscopy and histopathology.

Advancements in endoscopic technology have led to a steady increase in the detection rate of gastric polyps. In Western medicine, the primary approach involves endoscopic polyp electrocoagulation and resection, known for its simplicity and reduced patient discomfort. However, challenges persist, including polyp recurrence post-surgery and limited improvement in clinical symptoms in some cases.^[3,4]^ This recurrence may be attributed to the persistence of underlying pathological factors, such as phlegm and blood stasis, even after polyp removal. These factors can lead to the continuous formation of new polyps, necessitating repeated polypectomies under gastroscopy. These issues impose significant economic and psychological burdens on patients.

Endoscopic high-frequency electrocoagulation resection is the most common surgical procedure for gastric polyps. Nonetheless, there is ongoing debate regarding the necessity of painless gastroscopy during the procedure. In this study, 64 patients with gastric polyps from January to December 2021 were analyzed to evaluate the effectiveness of high-frequency electrocoagulation resection conducted under painless gastroscopy and its impact on the treatment of gastric polyps.

## 2. Information and methods

### 2.1. General information

This study was approved by the Ethics Committee of the Fifth People Hospital of Wuhu, 64 patients with gastric polyps treated by surgery in our hospital from January to December 2021 were segregated into 2 groups based on different treatment methods: an experimental group (32, G_E_) and a control group (32, G_c_). G_c_ patients received conventional medicine for 8 weeks, and then were treated with high-frequency electrocoagulation under conventional gastroscope; After 8 weeks of conventional drug treatment, G_E_ received high-frequency electrocoagulation under painless gastroscopy. In the control group, there were 12 males and 20 females with a maximum age of 80, minimum age of 44, and average age of 63.06 ± 9.51; The diameter of polyp was 0.2~2.2cm. In the experimental group, there were 9 males and 23 females; In the experimental group, maximum age was 78 years old, minimum age was 41 years old, and average age was 61.86 ± 9.48 years old; The diameter of polyp was 0.2 to 2.1cm.

Inclusion criteria: Diagnosed as gastric polyps by clinical gastroscopy. Meet the indications of surgical treatment. Medical records and other relevant data are clear and effective. All patients were informed and signed a letter of intent to consent. Patients over 18 years old and under 80 years old. Patients with polyp diameter ≤ 20mm. Exclusion criteria: Patients with important organ injuries. Have gastric cancer. Have other digestive tract diseases. Patients with coagulation dysfunction. Have a history of mental illness, or combined with communication and cognitive impairment. Poor treatment compliance, or those who do not agree to participate in the study. Familial polyposis. Pregnant or lactating women. Allergic constitution. Age < 18 years old or > 80 years old. Polyp diameter > 20mm or pedicle diameter > 10mm.

**Figure 1.**
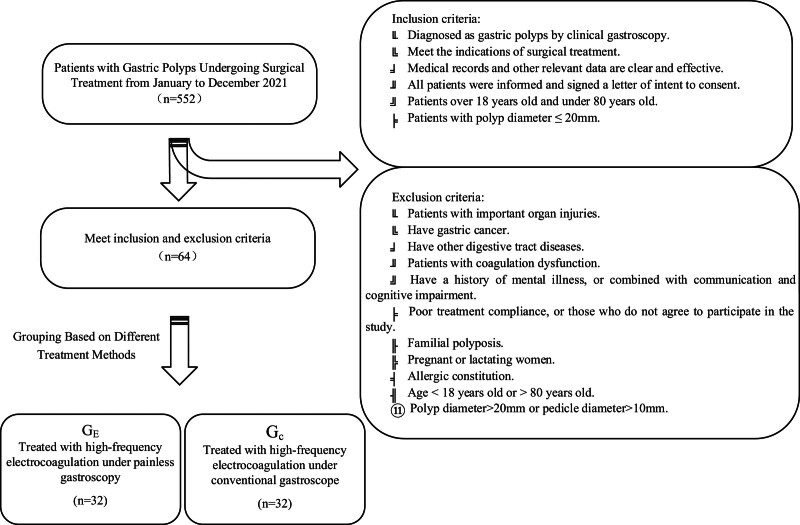
According to the inclusion and exclusion criteria, they were divided into control group and experimental group according to different treatment methods, 32 patients in each group.

Criteria for case exclusion and shedding: Patients who do not adhere to prescribed medication schedules, are unable to assess treatment efficacy, or have incomplete data can hinder the accurate evaluation of treatment effectiveness and safety. Patients who fell off naturally during the observation and were lost to follow-up. Serious adverse events and complications occurred during the test. Figure 1 shows a flow diagram of the inclusion and exclusion criteria.

### 2.2. Surgical methods

Experimental scheme: The experimental group was treated with conventional drugs for 8 weeks, and then treated with painless gastroscopy polyp high-frequency electrocoagulation and resection; The control group was treated with conventional drugs for 8 weeks, and then treated with high-frequency electrocoagulation and resection of polyps under ordinary gastroscope. High frequency electrocoagulation and resection method: find the target polyp under conventional endoscopy, and then place the polyp in the center of the field of vision and fully expose it by adjusting the gastroscope and changing the patient position direction. The distance between the polyp and the mirror end is generally 2 cm. If the volume is huge, it can be appropriate and far away. The snare device is inserted and the assistant is asked to open the snare loop. When holding the polyp, it is best to make the loop surface perpendicular to the polyp. The pedicled polyp is placed on the side of the pedicled polyp, and the sessile polyp is placed slightly above the base. The assistant gently and slowly closes and tightens the loop. After the set is well held, generally, the electric coagulation is adopted first, and then the electric cutting is adopted. The power is energized repeatedly and intermittently, or the mixed current can be used for intermittent power on. The power on time is several seconds each time, and it is gradually cut off. After resection, the polyp can be grasped and sucked by biopsy forceps or negative pressure suction method, and then withdrawn with the mirror body for pathological examination. For sessile polyps <0.5cm in diameter, electrocoagulation or hot biopsy forceps are generally used. The hot biopsy forceps cauterization method is suitable for relatively large sessile polyps. The polyp head is Hold with the hot biopsy forceps, and then gently pull the polyps upward to make the basement form a tentorial false pedicle. After the coagulation current is applied, the basement mucosa turns white, and then it can be extracted. The central tissue of the forceps will not be burned, so pathological examination can be done. Electrocoagulation and cauterization is suitable for polyps with smaller volume. An electrocoagulation device is inserted and the polyps are gently touched to electrify. The polyps are white and necrotic and can be cauterized. Postoperative precautions: stay in bed for 6h after operation, and avoid strenuous exercise within a week after operation; Fasting for 6h and liquid diet for 24h were observed after operation.

### 2.3. Outcome measures

Safety Observation Indexes: Comprehensive physical examination of patients; routine blood, urine, and stool tests; standard assessments of liver and kidney function; adverse reaction indicators. Efficacy Observation Indexes: Recording changes in symptoms before and after treatment using a symptom score quantization table, and evaluating scores of the primary symptoms. We quantified the symptoms and efficacy of patients before and after surgery using the Gastrointestinal Symptom Rating Scale questionnaire. Epigastric fullness, Epigastric pain, stunned without appetite, burn out and fatigue, nausea and vomiting, acid reflux belching, and Gastric noise were evaluated. Gastric Mucosal Healing Index: Observation of gastric mucosal healing in all cases using electronic gastroscopy at 2- and 4-weeks post-operation, and comparison of gastric mucosal healing between the 2 groups. Long-term Follow-up: Gastroscopy conducted 6 months after treatment completion to observe any recurrence of polyps.

### 2.4. Efficacy evaluation criteria

Cure criteria: The main clinical symptoms disappeared basically, and the gastroscopic polyps disappeared completely 4 weeks after operation, and the wound scar formed or disappeared. Effective standard: The main clinical symptoms were significantly improved, and the gastroscopic polyps disappeared completely 4 weeks after operation. The wound was basically healed, and there was still inflammation around, and the wound area was reduced by more than 50% of the original gastric mucosal injury. Invalid criteria: The main clinical symptoms had no obvious change or aggravation. Four weeks after operation, gastroscopy showed that there were still residual pedicle and residual uplift in the local mucosa. Histopathological examination confirmed that gastroscopy showed that the wound area was reduced by <50%. The cure standard of long-term curative effect: The polyp cured patients were reexamined by gastroscopy 6 months after the end of treatment, and no polypoid protrusion was found. The recurrence and cure criteria of long-term efficacy: The patients who were cured of polyps were reexamined by gastroscopy 6 months after treatment, and the polypoid protrusion of gastric mucosa was found, which was confirmed by histopathology.

### 2.5. Statistical methods

SPSS 22 statistical software analyzed measurement data. *T* test expressed measurement data in form of mean ± standard deviation. Data were counted by Chi square test. When *P* < .05 or *P* < .01, the data were significant.

## 3. Results

### 3.1. General data analysis

A total of 64 observation cases were selected, 2 shedding cases in G_E_, 2 shedding case in G_C_. 60 observation cases remained, with 30 cases in G_E_ and 30 cases in G_C_. The analysis results of general data are shown below.

Table 1 showed no difference in gender distribution, age distribution, and disease course distribution (*P* > .05).

**Table 1:** Analysis of general data results.

Gender	Male		Female		t	*P*
Experimental group	7 (23.3%)		23 (76.7%)		0.628	.28
control group	12 (40%)		18 (60%)			
Age (yr)	41–50	51–60	61–70	71–80	t	*P*
Experimental group	3 (10%)	8 (26.7%)	12 (40%)	7 (23.3%)	−0.476	.533
control group	2 (6.7%)	14 (46.7%)	7 (23.3%)	6 (20%)		
Course of Disease (mo)	0–6	6–12	12–24	>24	t	*P*
Experimental group	13 (43.3%)	8 (26.7%)	7 (23.3%)	2 (6.7%)	−0.680	.517
Control group	11 (36.7%)	9 (30%)	7 (23.3%)	3 (10%)		

### 3.2. Gastroscopy analysis before treatment in 2 groups

Table 2 shows results of gastroscopy and pathological examination. The results in Table 2 indicate that there are no differences in the diameter (T), morphology (Chi square), distribution (Chi square), number (Chi square), and pathological types (Chi square) of polyps (*P* > .05).

**Table 2:** Analysis of results of gastroscopy and pathological examination before treatment.

Pathological examination	Number	Type					Inspect	
Meatball diameter size (CM)	/	<0.5	0.5–1.0	1.1–1.5	1.6–2	/	t	*P*
Experimental group	30	3	24	2	1	/	1.655	.117
Control group	30	10	18	2	0			
Polyp morphology	Number	Yamada type I	Yamada type II	Yamada III type	Yamada IV type	/	X^2^	*P*
Experimental group	30	20	6	3	1	/	1.237	.758
Control group	30	21	4	2	3			
Distribution of polyp sites	Number	Gastric antrum	Gaussian body	Funds of stomach	Cardia	Gastric horn	X^2^	*P*
Experimental group	30	19	3	4	3	1	1.643	.814
Control group	30	16	5	7	1	1		
Distribution of polyp numbers	Number	Single shot	Pilosity	/			X^2^	*P*
Experimental group	30	10	20	/			0.311	.595
Control group	30	8	22					
Distribution of pathological types	Number	Infrared polyp	Hyperplastic polyp	Anonymous polyp	Functional gland polyp	/	X^2^	*P*
Experimental group	30	12	8	2	8	/	0.713	.884
Control group	30	14	6	3	7			

### 3.3. Main symptom score and total symptom score results before treatment

**Figure 2.**
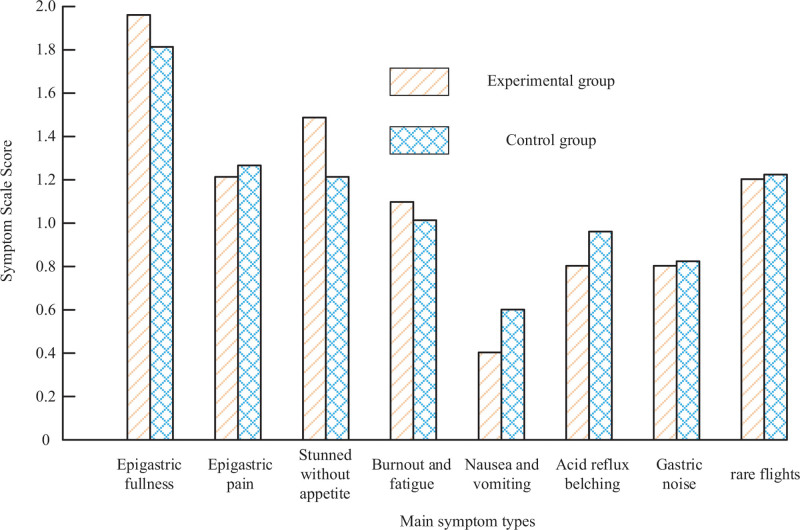
Results of main symptom scores and total symptom scores before treatment.

The main symptom score results of the 2 groups before treatment are shown in Figure 2. Figure 2 showed no difference in symptom scores for bloating, pain in the stomach, lack of appetite, fatigue, nausea and vomiting, noise in the stomach, acid reflux and belching, and loose stools (*P* > .05).

Table 3 shows total symptom scores results before treatment and no difference in their distributions (*P* > .05).

**Table 3:** Total symptom scores comparison of patients before treatment.

Project	Group		t	*P*
	Experimental group	Control group		
Symptom total points	9.14	8.98	0.151	.898
Variance	4.69	4.73		

### 3.4. Main symptom score results before and after treatment

**Figure 3.**
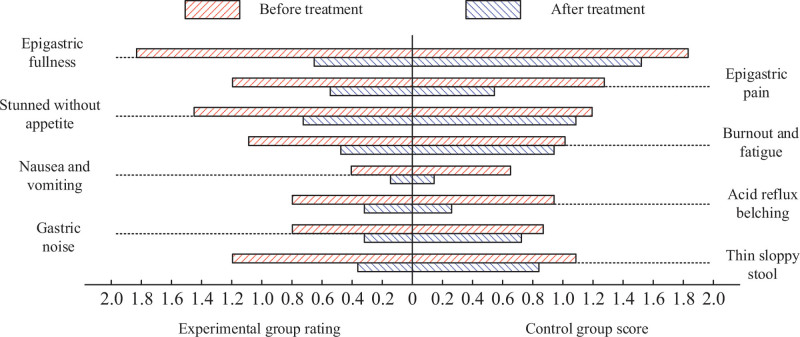
Score results of main symptoms before and after treatment.

Figure 3 shows main symptom scores differences before and after treatment (*P* < .01), and the score after treatment is lower, indicating that G_E_ method can improve main symptoms of patients with polyps; After treatment, the scores of epigastric pain, nausea and vomiting, acid regurgitation and belching in G_C_ were decreased after treatment (*P* < .01), indicating that control group improves symptoms in the above aspects. After treatment, symptom scores were different in the aspects of epigastric fullness, fatigue and loose stool (*P* < .01 or *P* < .05). G_E_ main symptoms score was lower, indicating the obvious advantages in improving the above symptoms compared with the control group.

Table 4 shows total symptom scores results differences before and after treatment (*P* < .01), and the total symptom score after treatment was significantly lower, indicating that the main polyps symptoms could be improved by methods adopted; G_E_ total score after treatment was lower, indicating the more advantages in improving total symptoms score of patients with polyps.

**Table 4:** Total symptom scores comparison before and after treatment.

Group	Number of cases	Total symptom score			
		Before treatment	Variance	After treatment	Variance
Experimental group	30	9.14	4.69	3.58*,#	3.32*^,^#
Control group	30	8.98	4.73	6.38*	4.04*

* Indicates that there is a significant correlation between 2 groups scores before and after treatment, *P* < .01.

# Indicates the comparison after treatment, *P* < .01.

Total score after treatment was lower, indicating that they could improve main gastric polyps symptoms (*P* < .01); G_E_ total score was lower after treatment, suggesting that G_E_ was better in improving total symptoms score in gastric polyps patients (*P* < .01).

### 3.5. Healing results of gastric mucosa in 2 groups

**Figure 4.**
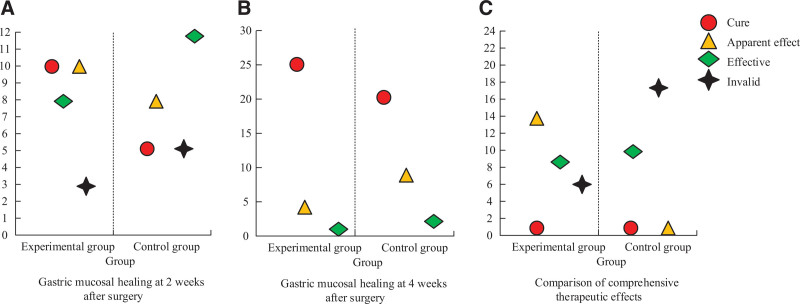
Results of gastric mucosal healing in 2 groups of patients.

The healing results of gastric mucosa in the 2 groups are shown in Figure 4. Figure 4A shows the results of gastric mucosal healing 2 weeks after operation. After rank sum test, gastric mucosal healing 2 weeks after operation showed a difference (*P* < .05). G_E_ cure rate was higher, indicating a better performance in gastric mucosal healing 2 weeks after operation. Figure 4B shows the results of gastric mucosal healing at 4 weeks after operation. After rank sum test, gastric mucosal healing at 2 weeks after operation showed no difference (*P* > .05). Figure 4C shows comprehensive curative effect results of 2 groups of patients. After rank sum test, the comprehensive curative effect results have a difference (*P* < .01). G_E_ efficiency and total effective rate are higher, indicating that G_E_ comprehensive curative effect is better.

Comparison of postoperative complications between the 2 groups. The experimental results revealed a lower recurrence rate in the GE group, with no recurrences observed in the experimental group within 6 months post-surgery. In contrast, the control group experienced 1 recurrence (1/28) at 3 months post-surgery and a total of 2 recurrences (2/28) at 6 months post-surgery. However, Fisher exact probability test did not show a significant difference (*P* > .05) (Table 5).

**Table 5:** Long term efficiency comparison.

Group	Number of follow up cases	Recurrence				Recurrence rate (%)
		6W-12W	13W-18W	19W-24W	Total (%)	
Experimental group	28	0	0	0	0	0
Control group	28	1	1	0	2	7.1

### 3.6. Operation results of 2 groups

**Figure 5.**
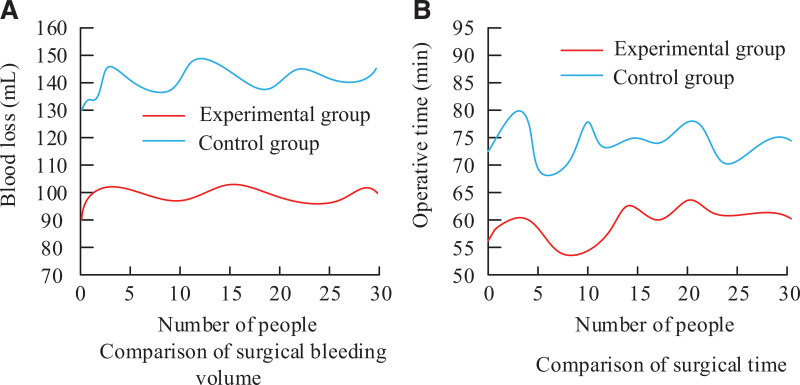
Surgical results.

Figure 5 shows the operation results. Figure 5A shows amount of bleeding in the operation. The results show that the amount of bleeding in G_E_ was significantly lower (*P* < .01). Figure 5B shows patients operation time. Results showed that G_E_ operation time was lower (*P* < .01).

### 3.7. Postoperative complications

Complications probability in both groups was low. Results showed that both methods had low probability of complications and the operation was safe. In the control group, there was 1 patient with gastric perforation (3.3%), 2 patients with postoperative upper abdominal pain (6.6%), and 1 patient with postoperative gastric hemorrhage (3.3%). In the experimental group, there was only 1 case of postoperative stomach pain (3.3%). Painless gastroscopy has a good trend in reducing the recurrence rate.

In this study, all cases had no drug-related adverse reactions after operation. There were no significant abnormal changes in routine functions.

## 4. Discussion

The exact causes and underlying mechanisms of gastric polyps remain incompletely understood. Many scholars believe that several factors may contribute to the development of gastric polyps, including long-term use of proton pump inhibitors (PPI), chronic gastritis, bile reflux, and genetic factors. Helicobacter pylori is a spiral gram-negative micro aerobic bacterium, which can be colonized on the gastric mucosal surface and mucosal layer. It is considered to be closely related to gastric cancer, peptic ulcer and other gastric diseases. The retrospective analysis of gastric polyps in China confirmed that inflammatory and proliferative polyps of the stomach were closely related to HP infection.^[5–7]^ The fifth National Consensus Report on the Treatment of Helicobacter pylori infection also lists proliferative gastric polyps as one of the indications for eradicating HP. Studies conducted abroad have shown that proliferative polyps can regress with HP eradication therapy, providing evidence that HP infection is a contributing factor in the development of certain types of gastric polyps. PPI has a strong acid inhibitory effect, which makes it widely used in treating digestive system diseases. Foreign studies have shown that the application time of PPI causes gastric fundus gland polyps, and has no obvious relationship with the dose of PPI.^[8–10]^ Currently, both domestic and international scholars generally agree that the use of PPI medications for more than 1 year is a relevant influencing factor for fundic gland polyps in the stomach. Research has demonstrated that the occurrence of proliferative gastric polyps is associated with chronic gastritis and autoimmune gastritis. Studies have also found a higher proportion of gastric adenomatous polyps, often coexisting with atrophic gastritis and intestinal metaplasia.^[11–13]^ Atrophic gastritis is considered a risk factor for gastric polyps. In the case of patients with gastric polyps and those with chronic gastritis without polyps, gastroscopic examinations have revealed that patients with gastric polyps are more prone to developing gastroesophageal reflux. This susceptibility may be related to the reflux of bile into the stomach, which can disrupt lysosomal membranes, dissolve lipoproteins, and compromise the integrity of the gastric mucosal barrier.^[14–16]^ In terms of genetics and other factors, studies found that gastric polyps occurrence and carcinogenesis connect mutations of tumor suppressor gene p53 and RAS, abnormal expression of tumor suppressor genes Ki67 and p16 and abnormal transduction pathways.^[17–19]^ Logistic regression analysis showed that gender and age were also risk factors for gastric polyps. In addition, smoking and heavy drinking will increase the risk of gastric polyps, which may be related to smoking will increase the infection rate of HP, heavy drinking can cause gastric mucosal damage and other factors.

The classification of gastric polyps at home and abroad is Morson histopathological classification, which divides polyps into neoplastic polyps and non neoplastic polyps. Neoplastic polyps include tubular adenoma, ductal papillary adenoma, papillary adenoma, and hereditary multiple polyposis. Non neoplastic polyps include hamartomatous polyps, inflammatory polyps, benign lymphofollicular polyps, and other metaplastic polyps.^[20–22]^ Patients with gastric polyps generally have no specific clinical manifestations, so the clinical diagnosis is mainly through auxiliary examination. The combination of electronic gastroscopy and biopsy is the gold standard to diagnose gastric polyps including endoscopic resection, surgical treatment and endoscopic combined drug therapy. At present, endoscopic resection is the main treatment, and high-frequency electrocoagulation is one of the main methods. The treatment of gastric polyps should not only select the appropriate resection method according to the location, size, shape, pedicle or non pedicle, number of polyps, but also consider the pathological type of gastric polyps.^[23–25]^

The occurrence of gastric polyps is caused by excessive hyperplasia of glands and gastric mucosal surface under the influence of related factors, resulting in wide base or papillary uplift with pedicle of gastric cavity. It is reported that the occurrence of adenomatous polyps is related to intestinal metaplasia, atrophic gastritis and other diseases. At the same time, gastric polyps are also a precancerous disease with a certain probability of canceration, so it needs to be removed after discovery. Endoscopic assisted high-frequency electrocoagulation resection is the main measure for the treatment of patients with gastric polyps. This operation has definite curative effect and less trauma. However, common painful endoscopic assisted surgery may induce postoperative complications such as perforation, ulcer, bleeding and abdominal pain, which has a certain impact on the prognosis.^[26–28]^ Studies found that occurrence of the above postoperative complications of patients is often related to the relatively low degree of intraoperative cooperation, so it is also necessary to explore effective improved surgical schemes to further alleviate the patients’ intraoperative pain and prevent postoperative complications.^[29,30]^ The application of painless gastroscopy is conducive to eliminating the fear of patients in the operation, and can effectively relieve the pain. Compared with ordinary gastroscopy, the application of painless gastroscopy is more humanized, which can better diagnose and treat patients’ diseases. By giving propofol injection, it is beneficial to reduce norepinephrine, and effectively block sympathetic nerve. At the same time, a certain negative inotropic effect appears on myocardium, which is beneficial to relieve cardiac relaxation and reduce intraoperative pain stimulation in reducing intraoperative stress response and improving comfort. The results of this study showed that the experimental group was treated with painless gastroscope assisted high-frequency electrocoagulation resection, which had better therapeutic effect. Symptom scores comparison after treatment showed that G_E_ had obvious advantages in improving gastric distension, fatigue and loose stool in patients with gastric polyps (*P* < .01 or *P* < .05). Total symptom score comparison after treatment showed that G_E_ was better (*P* < .01). G_E_ gastric mucosa healing was better at 2 weeks after operation (*P* < .05); No difference showed at 4 weeks after operation (*P* > .05). The comprehensive curative effect comparison showed that G_E_ was better (*P* < .01). No difference showed in long-term efficacy (*P* > .05). And there is no recurrence of patients after operation, and the probability of complications is also low, which proves that painless gastroscopy assisted high-frequency electrocoagulation resection can better improve the safety of clinical treatment of patients with gastric polyps, and ensure the therapeutic effect. In conclusion, painless gastroscopy assisted high-frequency electrocoagulation resection can obtain satisfactory curative effect and high safety in treating gastric polyps.

## Author contributions

**Conceptualization:** Xiaomei Chen, Mei Chen.

**Data curation:** Dandan Zhang.

**Formal analysis:** Xiaomei Chen.

**Investigation:** Mei Chen.

**Methodology:** Xiaomei Chen.

**Supervision:** Mei Chen.

**Writing – original draft:** Xiaomei Chen.

**Writing – review & editing:** Xiaomei Chen, Mei Chen.
